# Genetic Relationships Between Residual Gain and Economically Important Traits in Nellore Cattle

**DOI:** 10.1111/jbg.70050

**Published:** 2026-04-09

**Authors:** Miller Teodoro, Gabriel Gubiani, Letícia Pereira, Amanda Santos Gubiani, Angélica Simone Cravo Pereira, Roberto Sainz, Cláudio Ulhôa Magnabosco, Fernando Baldi

**Affiliations:** ^1^ Department of Animal Science São Paulo State University—Júlio de Mesquita Filho (UNESP) Jaboticabal Brazil; ^2^ Department of Veterinary Medicine, Faculty of Animal Science and Food Engineering University of São Paulo Pirassununga Brazil; ^3^ Sustainable Livestock Research Center, Animal Science Institute São José do Rio Preto Brazil; ^4^ Department of Nutrition and Animal Production, Faculty of Veterinary and Animal Science University of São Paulo Pirassununga Brazil; ^5^ Department of Animal Science University of California Davis California USA; ^6^ Brazilian Agricultural Research Corporation (Embrapa Cerrados) Planaltina Federal District Brazil

**Keywords:** beef cattle, feed efficiency, sustainability

## Abstract

Sustainable beef production requires identifying animals with superior feed efficiency to reduce environmental impact and production costs. This study aimed to estimate heritability and genetic correlations between residual gain (RG) and growth, reproductive, carcass and feed efficiency traits in Nellore cattle. A total of 217,333 phenotypic records from animals born between 1980 and 2024, raised in diverse Brazilian regions, were analysed using Bayesian inference with a multi‐trait mixed animal model. Heritability estimates for reproductive traits were low, while feed efficiency, growth and carcass traits showed moderate heritabilities. Residual gain exhibited a moderate positive genetic correlation with adjusted weight at 450 days (W450), supporting its potential as a selection criterion for growth efficiency. Genetic correlations between RG and carcass, reproductive and feed efficiency traits were generally low or near zero. However, a negative low genetic correlation and a moderate positive phenotypic correlation with early conception probability indicate complex effects on reproduction. Selection for RG may increase yearling weight and maintenance energy requirements, which can reduce early reproductive performance under restricted nutritional conditions, presenting trade‐offs to consider in extensive systems. Conversely, RG may be particularly suitable for intensive systems such as feedlot or finishing operations, where nutritional management can mitigate these limitations. The negative genetic correlation between RG and residual feed intake further highlights RG's ability to identify animals that grow efficiently without increased feed intake. These results confirm that RG is a largely independent trait and shows sufficient genetic variability to respond to selection in Nellore cattle. Using it as a selection criterion can enhance feed efficiency without negatively impacting other economically important traits.

## 1 Introduction

Advancing sustainable animal protein production has become a global priority, driven by a projected world population of 10.3 billion and a 12% rise in meat production by 2033 (United Nations 2024; OECD/FAO 2024). Meeting this demand requires improving productivity in tropical and subtropical regions, which host over 80% of the global cattle population (Cooke et al. 2020). *Bos indicus* cattle, such as the Nellore breed, are crucial due to their adaptability to heat and nutritional constraints (Scholtz et al. 2013). Thus, selecting for feed efficiency is essential to identify animals that convert feed into edible meat more effectively, reducing production costs and enteric methane emissions while ensuring food security (Berry and Crowley 2013; Brunes et al. 2021; Pereira et al. 2023; Sainz et al. 2024).

Estimates of genetic parameters, such as heritabilities and genetic correlations, are essential to support selection decisions in breeding programmes that balance productive efficiency with environmental sustainability (Lynch and Walsh 1998; Falconer and Mackay 2009; Mrode 2014). While traditional traits have been extensively studied (Meyer et al. 1993; Mota et al. 2019; Srivastava et al. 2019), attention must be directed by modern breeding programmes toward the genetic architecture of feed efficiency and its association with growth, carcass and reproductive traits, including sexual precocity and longevity (Ceacero et al. 2016; Brunes et al. 2021). Understanding these additive genetic relationships is fundamental to producing beef more efficiently while minimizing methane production.

Among feed efficiency indicators, residual feed intake (RFI) is widely used to identify animals that consume less feed than expected for their maintenance and tissue deposition (Koch et al. 1963; Crews Jr. 2005). Because of its moderate heritability and independence from growth traits, selection for lower RFI in Nellore cattle has shown positive results, reducing maintenance energy costs and enteric methane emissions without compromising adult size (Ceacero et al. 2016; Sainz et al. 2024). These outcomes reinforce the utility of RFI as a criterion for more sustainable beef production. However, since RFI reflects deviations in consumption rather than improvements in productive output, it may be less effective in identifying animals with superior biological efficiency in terms of weight gain and time to slaughter.

As an alternative, residual gain (RG) quantifies the difference between observed weight gain and that predicted based on feed intake. This metric enables the identification of animals that convert feed into growth more efficiently, regardless of their absolute intake or body size. Animals with positive RG exhibit greater‐than‐expected weight gain without a corresponding increase in feed consumption, aligning with the ideal profile of both biological and economic efficiency (Berry and Crowley 2013). In Nellore cattle, RG has shown favourable genetic correlations with growth and carcass traits (Magnabosco et al. 2020; Brunes et al. 2024), reinforcing its potential as a strategic metric. By offering greater precision in identifying animals that combine high feed efficiency with superior performance, improving RG may lead to faster growth rates and shorter time to slaughter (Idowu et al. 2024).

The use of the RG trait promotes the development of more resilient and sustainable production systems, aligned with the demands of the global market and contemporary environmental challenges (Scholtz et al. 2013; Magrin et al. 2014). While Brunes et al. (2021) investigated RG in Nellore cattle, their study was based on a limited dataset that did not include reproductive traits, accentuating the need for further research to assess the broader genetic implications of RG, particularly on fertility and longevity. Therefore, understanding the genetic parameters associated with RG and their correlations with economically important traits is essential to support more effective selection strategies in beef cattle production. The present study aimed to estimate the heritability and genetic correlations of residual gain with growth, carcass, feed efficiency and reproductive traits in Nellore cattle, thereby supporting genetic improvement programmes focused on enhancing productive efficiency and the sustainability of beef production systems.

## 2 Materials and Methods

### 2.1 Ethics Statement

This study was exempt from evaluation by the Animal Ethics Committee (CEUA), as established by Law No. 11,794 of 08/10/2008 and Normative Resolution No. 51 of 05/19/2021 from the National Council for Animal Experimentation Control (CONCEA) because the data were obtained from an existing database.

### 2.2 Phenotypic Data

The data used in this study is part of the animal breeding programme for Nellore cattle in Brazil, which is coordinated by the National Association of Breeders and Researchers (ANCP) in Ribeirão Preto, Brazil. The dataset includes records on growth, reproductive, carcass and feed efficiency traits, which are considered economically important in Brazilian beef production. A total of 217,333 phenotypic records were collected from Nellore animals born between 1980 and 2024. These animals originated from 675 ANCP partner herds located across Brazil's Central‐West, Southeast, Northeast and North regions. The pedigree contained information on 411,892 animals, overlapping three generations, comprising 17,784 sires and 216,196 dams.

### 2.3 Traits

The traits related to growth included adjusted weight at 240 days (W240), adjusted weight at 450 days (W450), adult weight (AW) and frame score (FRAME). Standardized weights were estimated using linear regression, based on the average daily gain (ADG) between 165 and 255 days for W240 and between 405 and 495 days for W450 (Negreiros et al. 2022). For AW, the cow's weight at weaning, closest to 5 years of age, within the 4.5 to 5.5‐year interval, was used (Boligon et al. 2009). FRAME is a quantitative index (ranging from 1 to 12) used to characterize skeletal development and growth capacity in cattle. In Nellore animals, FRAME values were estimated using a predictive equation that integrates hip height, age and ultrasound traits, enabling standardization of animals based on expected carcass weight and fat deposition under Brazilian production systems (Negreiros et al. 2022; Sainz et al. 2023). Frame score is often evaluated alongside liveweight and other performance traits to support herd management decisions, aligning nutritional requirements with feed resources and production objectives. Large‐frame cattle typically grow faster, have a higher muscle‐to‐fat ratio, produce leaner carcasses and reach puberty later, but also exhibit greater mature size and higher maintenance‐energy demands. Conversely, moderate‐ to low‐frame animals tend to be more efficient in extensive, low‐input systems common in tropical and subtropical regions, where environmental variability and limited resources favour sustainable production (Negreiros et al. 2022).

Carcass traits were measured via ultrasound, as follows: REA, BFT and MAR: *Longissimus dorsi* area, subcutaneous fat thickness and % intramuscular fat, respectively, between the 12th and 13th ribs; RFT: rump fat thickness between ilium and ischium (intersection of *Gluteus medius* and *Biceps femoris*). Ultrasound evaluations were performed by technicians certified by the Brazilian Association of Ultrasound Technicians (ATUBRA) and the Ultrasound Guidelines Council (UGC), following Beef Improvement Federation (BIF 2018) and Ultrasound Guidelines Council (UGC) guidelines.

Longevity was assessed using the stayability (STAY) trait. Cows that produced at least three calves by 76 months of age were considered successful (score 2), while those that did not reach this threshold were considered failures (score 1). Reproductive traits were evaluated using the early calving probability (ECP), age at first calving (AFC) and cumulative productivity (CP) for females, and scrotal circumference at 365 days (SC365) for males. SC365 was measured using a scrotal measuring tape at 3‐month intervals from 9 to 18 months of age, followed by linear adjustment to 365 days. For ECP, heifers were challenged for sexual precocity (heifers who become pregnant by 30 months), entering the first breeding season at 11 months of age, minimum weight of 250 kg, with successful cases receiving a score of 2 and failures a score of 1. The AFC was recorded as the dam's age in months at her first calving. The CP trait, proposed by Schwengber et al. (2001) as a selection criterion for Nellore, indicates the productivity of the dam; it is calculated as the total weaning weight of all calves produced per year by a dam. Also, it evaluates the precocity and reproductive periodicity of these same dams, including their maternal abilities to wean heavier calves, as shown in the following equation (Grossi et al. 2008):

(1) $$\mathrm{CP}=W_{w}\times n_{p}\times \frac{365}{{\mathrm{ACB}}_{n}},$$

which $W_{w}$ is the average weight of calves at weaning; $n_{p}$ is the total number of calves produced; and ${\mathrm{ACB}}_{n}$ is the age of the cow at the last birth. Therefore, CP constitutes a comprehensive and suitable selection criterion for use in herd breeding, as it encompasses both productive and reproductive traits.

The feed efficiency traits used were the residual feed intake (RFI), dry matter intake (DMI) and residual gain (RG), obtained through Intergado and GrowSafe electronic systems. Feed efficiency tests followed the guidelines established by Mendes et al. (2020), for assessing individual feed intake in beef cattle using both electronic systems. Animals were kept in collective or individual pens and subjected to a 7‐day (animals from the same herd) or 21‐day (lots from different herds) adaptation period followed by a valid testing phase. The testing phase included at least 35 valid daily feed intakes and at least 42 valid daily body weights, subject to additional precision criteria (standard error mean no greater than 5% and 31% of the mean for DMI and ADG, respectively). Throughout this period, each animal's average weight was recorded via manual weighing every 14 days or through automated weighing platforms (Intergado).

The RFI and RG are defined as conditional traits derived from regression models that represent the distribution of feed intake or gain conditional on metabolic body weight (MW^*0.75*^) and performance‐related variables. Although this classical two‐step approach ensures independence between the residual trait and the conditioning variables at the phenotypic level, such independence is not necessarily guaranteed at the genetic level, and estimates may be influenced by the distribution of fixed effects across contemporary groups (CG), as observed in recent studies addressing genetic‐level independence (Esfandyari and Jensen 2021). Nevertheless, the regression‐based formulation originally proposed by Koch et al. (1963) remains the most widely adopted approach in feed efficiency research and genetic evaluation programmes, providing a practical, standardized framework for calculating residual efficiency traits.

To obtain RFI, the ADG and MW^*0.75*^ were calculated. Daily DMI was derived from the means of all valid individual daily intake values electronically recorded by the Intergado and GrowSafe systems during the test period, multiplied by the mean dry matter percentage throughout each test. The ADG were estimated using the linear regression coefficient of weight against days on test for the animals (DOT) using the *lm* function in the R program with the following equation (Koch et al. 1963):

(2) $$y_{i}=\alpha +\beta {\mathrm{DOT}}_{i}+\varepsilon_{i},$$

where $y_{i}$ is the animal's weight, α is the intercept of the regression equation that represents the initial weight, β is the linear regression coefficient that represents ADG, ${\mathrm{DOT}}_{i}$ are the days in the performance test of the *n*th observation, and $\varepsilon_{i}$ is the error associated with each observation.

The mean MW^0.75^ was calculated using the equation below (Koch et al. 1963):

(3) $${\mathrm{MW}}^{0.75}={\left[\alpha +\beta \left(\frac{{\mathrm{DOT}}_{j}}{2}\right)\right]}^{0.75},$$

where α is body weight at the beginning of the feed efficiency test, β is the ADG and ${\mathrm{DOT}}_{j}$ is days on test.

The RFI was calculated as the difference between predicted and observed dry matter intake, using a regression equation based on MW^0.75^ and ADG, following the methodology proposed by Koch et al. (1963):

(4) $$y=\beta_{0}+\beta_{1}\mathrm{ADG}+\beta_{2}{\mathrm{MW}}^{0.75}+\varepsilon \left(\mathrm{RFI}\right),$$

where y is the individual DMI of the animal, $\beta_{0}$ is the intercept, $\beta_{1}$ and $\beta_{2}$ are the linear regression coefficients for ADG and MW^0.75^, and ε is the residual error, which is equivalent to the RFI.

The RG was calculated as the difference between the observed ADG and the estimated based on MW^0.75^ and DMI (Koch et al. 1963; Berry and Crowley 2013):

(5) $$Y=\beta_{0}+\beta_{1}\mathrm{DMI}+\beta_{2}{\mathrm{MW}}^{0.75}+\varepsilon \left(\mathrm{RG}\right),$$

where $\beta_{0}$ is the intercept, $\beta_{1}$ and $\beta_{2}$ are the linear regression coefficients for DMI and MW^0.75^, and ε is the residual error, which is the RG in this case.

For the growth, reproductive and carcass‐related traits, the CG was composed of herd, year, birth season (dry season: April to September, rainy season: October to March), sex and management group at weaning and yearling. For RFI, RG and DMI, the CG was composed of herd, management group, feed efficiency test, sex, year and birth season and the animal age at the beginning of the test as a linear covariate. Due to the long breeding season, births spanned the dry and wet seasons. Records that stood out by 3.5 standard deviations from the CG mean were removed. For feed efficiency traits, CG with fewer than five animals were removed. For other traits, CG with fewer than 15 and more than 200 animals were excluded to retain only the most informative animals. For the categorical variables STAY and ECP, groups with zero variance were also removed from the analyses. A summary of the dataset used is shown in Table 1.

**TABLE 1:** Description of the dataset for key traits in Nellore cattle.

Traits	Animals	CG	Mean	SD	Min	Max
RG (kg/day)	27,101	683	0	2.07	−1.50	1.90
RFI (kg of DM/day)	27,110	683	0	0.68	−5.40	4.40
DMI (kg/day)	27,496	683	8.50	0.19	2.50	20.70
W240 (kg)	26,735	255	194.38	34.33	67	317
W450 (kg)	27,273	236	302.60	63.85	128	586
AW (kg)	25,517	722	507.42	79.32	230	1060
FRAME (points)	25,959	427	5.90	2.05	1	12
RFT (mm)	25,992	665	6.33	2.83	0.51	22.22
BFT (mm)	25,948	663	3.92	1.93	0.45	19.56
MAR (%)	24,940	829	2.75	1.00	0.17	12.70
REA (cm^2^)	25,995	664	54.64	8.92	18.59	94.00
STAY (2 success, 1 failure)	25,408	366	1.44	0.50	1	2
ECP (2 success, 1 failure)	25,259	448	1.46	0.50	1	2
SC365 (cm)	27,077	311	22.15	2.78	12.90	33.20
AFC (months)	25,281	395	31.26	6.25	19	49
CP (kg of calf weaned/cow/year)	24,856	439	166.97	33.06	44	302

Abbreviations: AFC, age of first calving; AW, adult weight; BFT, backfat thickness; CP, cumulative production; DMI, dry matter intake; ECP, early calving probability; FRAME, frame score; MAR, marbling; REA, ribeye area; RFI, residual feed intake; RFT, rump fat thickness; RG, residual gain; SC365, adjusted scrotal circumference at 365 days of age; STAY, stayability; W240, adjusted weight at 240 days of age; W450, adjusted weight at 450 days of age.

### 2.4 Genetic Parameters Estimation

Genetic parameters were estimated using multi‐trait mixed animal models based on pedigree information. A total of 16 traits were evaluated. Three separate multi‐trait analyses were conducted to estimate genetic and phenotypic correlations between RG and each of the other evaluated traits, using RG, DMI, RFI and AW as anchor traits, grouping additional traits according to growth, carcass, and reproductive categories. (Co)variances were estimated using Bayesian inference via the GIBBSF90+ software, part of the BLUPF90 family of programmes (Misztal et al. 2002), as follows:

(6) $$y=\mathrm{X\beta}+Z_{1}u_{d}+Z_{2}u_{m}+Z_{3}p_{m}+e,$$

where y is the vector of phenotypic records, β is the vector of systematic effects, *X* is the incidence matrix associating β with *y*, $u_{d}$ is the vector of direct additive genetic effects, $Z_{1}$ is the incidence matrix associating $u_{d}$ with *y*, $u_{m}$ is the vector of maternal additive (mat) effects, $Z_{2}$ is the incidence matrix associating $u_{m}$ with *y*, $p_{m}$ is the vector of permanent maternal (mpe) effects, $Z_{3}$ is the incidence matrix associating $p_{m}$ with *y*, and *e* is the residual effect. The mat and mpe effects were fitted only for W240 and omitted for other traits. The CG and dam age at calving (DAC) were fitted as systematic effects, with DAC included for all traits except AFC, CP and FRAME. For carcass and efficiency traits, the model also included linear and quadratic effects of animal age as covariates.

A threshold model was applied for ECP and STAY, assuming that the underlying tendency follows a standard normal distribution, as described by Mrode (2014): $U\left|\theta \sim N\left(\theta ,I\right.\right.\left.\sigma_{e}^{2}\right)$, where *U* is the vector of latent (underlying) liabilities with dimension *r* × *r* (number of animals), θ = (β, $u_{d}$) is the vector of effects conditioned on in the model, including fixed effects (β) with dimension *s* and direct additive genetic effects ($u_{d}$), *I* is the identity matrix of order *r* × *r* and $\sigma_{e}^{2}$ is the residual variance, fixed to 1 for binary traits, following standard threshold model assumptions. The same model as in Equation (6) was used, excluding mat and mpe effects, with CG included for STAY and CG and DAC included for ECP.

All random effects were assumed to follow multivariate normal distributions. In all analyses, RG, DMI, RFI and AW were included as anchor traits. The first multi‐trait analysis combined these traits with growth traits (W240, W450 and FRAME), whereas the second combined them with carcass traits (RFT, REA, BFT and MAR). Reproductive traits were evaluated in two separate multi‐trait analyses: one including SC365, STAY, ECP and CP, and another including SC365, STAY, AFC and CP, because the joint inclusion of ECP and AFC in the same multi‐trait Bayesian model resulted in a lack of convergence and poor mixing of the Markov chains. For all analyses, random effects were assumed to be distributed as: $u_{d}\sim \mathrm{MVN}\left(0,A⨂G_{0}\right)$, $u_{m}\sim \mathrm{MVN}\left(0,A⨂G_{1}\right)$, $p_{m}\sim \mathrm{MVN}\left(0,I⨂G_{2}\right)$ and $e\sim \mathrm{MVN}\left(0,I⨂R_{0}\right)$, where A is the numerator relationship matrix, I is the identity matrix, and $G_{0}$, $G_{1}$, $G_{2}$ and $R_{0}$ are the (co)variance matrices for direct additive genetic, mat, mpe and residual effects, respectively, as follows:

$$G_{0}=\left[\begin{array}{ccc} \sigma_{u_{d1}}^{2} & \cdots & \sigma_{u_{d1n}}\\ \vdots & \ddots & \vdots \\ \sigma_{u_{\mathrm{dn}1}} & \cdots & \sigma_{u_{\mathrm{dn}}}^{2} \end{array}\right];R_{0}=\left[\begin{array}{ccc} \sigma_{e_{1}}^{2} & \cdots & \sigma_{e_{1n}}\\ \vdots & \ddots & \vdots \\ \sigma_{e_{n1}} & \cdots & \sigma_{e_{n}}^{2} \end{array}\right]$$

The mat and mpe effects ($G_{1}$ and $G_{2}$) were included only for W240 and all other elements were set to zero. The order of the matrices (*n*) varies depending on the number of traits included in each multi‐trait analysis. The phenotypic vector y was assumed to follow:

*y* ∣ β, $u_{d}$, $u_{m}$, $p_{m}$, $G_{0}$, $G_{1}$, $G_{2}$∼ *N* ($\mathrm{X\beta}+Z_{1}u_{d}+Z_{2}u_{m}+Z_{3}p_{m}$, $R_{0}$), and $\sigma_{u_{\mathrm{di}}}^{2}$, $\left(\sigma_{u_{\mathrm{dij}}}\right)$, $\sigma_{u_{\mathrm{mi}}}^{2}$, $\left(\sigma_{u_{\mathrm{mij}}}\right)$, $\sigma_{p_{m1}}^{2}$, $\left(\sigma_{p_{m12}}\right)$, $\sigma_{\mathrm{ei}}^{2}$, $\left(\sigma_{\mathrm{eij}}\right)$, where *i*, *j* = 1 for RG and 2 for the second trait, represent the respective direct additive genetic, mat, mpe and residual (co)variances. An inverse scaled Wishart distribution with non‐informative hyperparameters was used as the prior for all (co)variance matrices, and a flat (non‐informative) prior was assumed for the fixed effects vector β. Covariances between direct and maternal genetic effects were not modelled.

The Gibbs sampling chain was run for 1,000,000 iterations, with a burn‐in of 50,000 iterations and a sampling interval of 100. Genetic parameters, including (co)variances and correlations, were estimated based on the posterior samples from all retained iterations, and the posterior means were used as point estimates. Convergence of the Markov Chain Monte Carlo (MCMC) chains was assessed using the R package coda, which provides diagnostics for MCMC output (Plummer et al. 2006). Heritability estimates were classified as low (< 0.20), moderate (0.20–0.40) or high (> 0.40) (Bourdon 2000). Genetic and environmental correlations were classified as low (< 0.30), moderate (0.30–0.70) or high (> 0.70) Hill (2013).

## 3 Results and Discussion

The analysis of genetic parameter estimates (Table 2) indicates that the expression of RG is predominantly influenced by environmental factors, as reflected in its low heritability. The heritability estimate for RG in this study aligns with previous findings in Nellore cattle, such as those by Brunes et al. (2021) and Ceacero et al. (2016), who reported heritability of 0.17 and 0.19, respectively, both using fewer RG records than those used in the present study. Although low heritability might suggest a limited direct response to selection, it does not diminish the economic importance of the trait. Complex traits, such as RG, involve intricate biological processes and often exhibit low heritability. However, it can still show meaningful genetic progress when modern selection tools are applied. The incorporation of genomic selection, multi‐omics data and systems biology approaches can enhance the identification of genetically superior animals and accelerate gains for such traits. Despite its low heritability, RG remains a biologically meaningful indicator of feed efficiency, as it identifies animals with greater‐than‐expected weight gain relative to feed intake, independent of body size or absolute intake. This provides a practical advantage in selecting animals that combine growth potential with efficiency.

**TABLE 2:** Posterior means and, between parentheses, the 95% highest density interval of genetic parameters for economically important traits in Nellore cattle.

Traits	$h^{2}$	$h_{m}^{2}$	$r_{g}$	$r_{p}$
RG	0.11 (0.08; 0.14)			
RFI	0.16 (0.13; 0.19)		−0.24 (−0.40; −0.08)	−0.30 (−0.31; −0.29)
DMI	0.31 (0.27; 0.35)		0.14 (0.00; 0.30)	0.01 (0.00; 0.03)
W240	0.23 (0.20; 0.25)	0.09 (0.08; 0.10)	0.21 (0.06; 0.36)	0.00 (−0.06; 0.06)
W450	0.34 (0.30; 0.38)		0.32 (0.10; 0.50)	0.00 (−0.05; 0.05)
AW	0.25 (0.21; 0.28)		0.28 (0.09; 0.48)	0.01 (−0.08; 0.10)
FRAME	0.46 (0.42; 0.51)		0.18 (0.00; 0.35)	0.02 (−0.02; 0.05)
RFT	0.42 (0.38; 0.46)		0.17 (0.00; 0.33)	0.01 (−0.06; 0.08)
BFT	0.33 (0.29; 0.37)		−0.08 (−0.23; 0.08)	−0.04 (−0.10; 0.03)
MAR	0.34 (0.31; 0.38)		−0.02 (−0.17; 0.13)	0.00 (−0.09; 0.08)
REA	0.36 (0.32; 0.40)		0.05 (−0.13; 0.24)	−0.04 (−0.10; 0.02)
STAY	0.19 (0.15; 0.23)		0.03 (−0.27; 0.30)	0.02 (−0.12; 0.15)
ECP	0.18 (0.13; 0.22)		−0.27 (−0.67; 0.11)	0.55 (0.46; 0.64)
SC365	0.44 (0.39; 0.48)		−0.03 (−0.21; 0.15)	−0.08 (−0.10; −0.04)
AFC	0.13 (0.08; 0.17)		−0.03 (−0.42; 0.28)	0.01 (−0.16; 0.17)
CP	0.11 (0.08; 0.1)		−0.20 (−0.51; 0.12)	−0.02 (−0.05; 0.01)

Abbreviations: AFC, age of first calving; AW, adult weight; BFT, backfat thickness; CP, cumulative production; DMI, dry matter intake; ECP, early calving probability; FRAME, frame score; *h* ^*2*^, heritability; *h* ^*2*^_*m*_, maternal heritability; MAR, marbling; REA, ribeye area; RFI, residual feed intake; RFT, rump fat thickness; *r*_*g*_, genetic correlation; RG, residual gain; *r*_*p*_, phenotypic correlation; SC365, adjusted scrotal circumference at 365 days of age; STAY, stayability; W240, adjusted weight at 240 days of age; W450, adjusted weight at 450 days of age.

The heritability estimates for other traits varied across categories (Table 2). Estimates were low for RFI, STAY, ECP, AFC and CP, moderate for DMI, W240, W450, AW, BFT, MAR and REA, and high for FRAME, RFT and SC365. These values are consistent with reports in Nellore cattle from previous studies (Yokoo et al. 2010; Caetano et al. 2013; Grion et al. 2014; Lopes et al. 2016; Pires et al. 2017; Kluska et al. 2018; Bonamy et al. 2019; Negreiros et al. 2022; Brunes et al. 2023; Carvalho et al. 2023; Sainz et al. 2024; Teodoro et al. 2025). Comparable heritability estimates were also reported in Guzerat cattle by Pereira et al. (2023), reinforcing the consistency of these parameters across Zebu breeds and supporting their utility in selection programmes in tropical systems. The maternal heritability estimates of W240 were low, as reported by Oliveira et al. (2021), Silveira et al. (2024) and Teodoro et al. (2025), in Nellore cattle. Although the maternal effect estimates were low, its inclusion in the model for W240 is recommended. Omitting this effect may result in overestimated direct heritability (Koetz Junior et al. 2019).

Heritability estimates in this study for the feed efficiency traits RFI and DMI were within the ranges reported for beef cattle for RFI (0.13–0.33) and DMI (0.25–0.36) (Grion et al. 2014; Oliveira et al. 2014; Olivieri et al. 2016; Silva et al. 2016; Moraes et al. 2017). These estimates indicate the potential for achieving genetic gains through selection focused on feed efficiency traits (Arthur et al. 2001; Bonamy et al. 2019).

The genetic correlations between RG and growth traits were positive, being low for W240, AW and FRAME, and moderate for W450. The favourable association with W450 suggests that selecting higher RG would improve yearling weight. Animals with higher RG demonstrate greater feed conversion efficiency, allowing surplus energy to be directed toward growth and development. Although the genetic correlation with AW is low (0.28), sustained selection for RG over multiple generations may gradually increase adult weight. Larger adult size can increase maintenance energy requirements, which may be disadvantageous in production systems with limited feed resources or seasonal shortages. Higher energy demand can reduce resilience and overall efficiency, potentially negatively impacting reproductive performance and calf weaning weight due to reduced energy available for reproduction and lactation. These trade‐offs could partially offset the benefits of improved growth. However, in intensive systems such as feedlots or finishing operations, where nutritional resources and management are more controlled, the higher maintenance energy requirements of larger animals may be less limiting. Therefore, selection for RG could be particularly suitable and effective in these environments, allowing producers to capitalize on improved growth and feed efficiency without the constraints often faced in extensive or resource‐limited systems. These results reinforce the relevance of RG as a complementary selection criterion in breeding programmes, in agreement with previous studies such as Ceacero et al. (2016). The genetic correlations of RG with carcass traits were low, indicating that improving RG would not affect carcass traits in Nellore cattle. This supports earlier findings by Santana et al. (2014), Ceacero et al. (2016), and Sainz et al. (2024), which highlight the genetic independence of RG from these traits.

**FIGURE 1:**
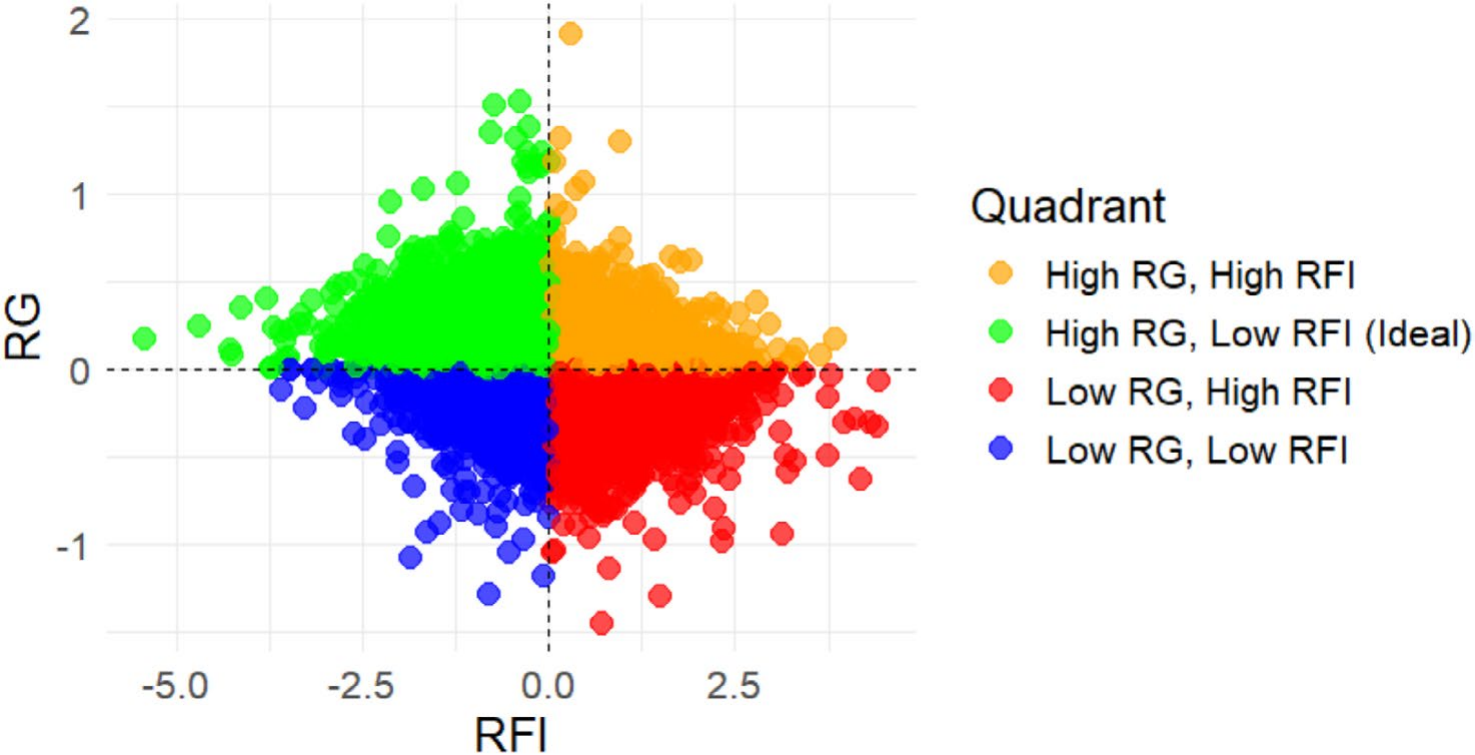
Classification of animals based on residual gain (RG) and residual feed intake (RFI) showing quadrants of growth and feed efficiency performance. [Colour figure can be viewed at wileyonlinelibrary.com]

The genetic correlations of RG with RFI and DMI were low, being negative with RFI and positive with DMI. This pattern is expected, given that both traits are defined as conditional traits, as described in the Materials and Methods, and adjusted for feed intake, indicating that animals genetically predisposed to higher RG tend to consume more feed. According to nutritional energetics, as intake increases, the proportion of ingested energy used for maintenance decreases, resulting in more energy available for tissue deposition. Nonetheless, the low genetic correlation between RG and DMI suggests that animals with higher RG do not necessarily consume substantially more feed, which is advantageous economically and environmentally. Ideally, producers should seek animals that combine superior growth (high RG) with better feed efficiency (low RFI), targeting individuals that perform well for both traits. Evaluating RG and RFI jointly can improve the identification of animals with high growth potential and efficient feed utilization. This relationship is illustrated in Figure 1, where animals are classified into four quadrants based on their RG and RFI values, highlighting the ideal group (green) with high RG and low RFI. This complementary approach is supported by studies in taurine and zebu populations (Sobrinho et al. 2011; Berry and Crowley 2013), which highlight the genetic relationship among intake, RFI and RG. Regarding reproductive traits, the genetic correlations between RG and STAY, AFC, CP and SC365 were low, suggesting that selection for higher RG would not negatively impact reproductive performance in either males or females. The genetic correlation between RG and ECP is also low; it is negative and close to moderate (−0.27), indicating that selection for high RG could, over time, reduce ECP. Overall, these results suggest that selection for RG can be applied without major adverse genetic effects on reproductive traits. However, it is necessary to monitor ECP when aiming for reproductive performance.

The phenotypic correlations of RG with growth, carcass, feed efficiency and reproductive traits were generally low. This suggests that the joint influence of additive genetic and environmental effects linking these traits is limited. However, a moderate positive correlation with ECP suggests that factors such as nutrition and management significantly affect their expression. These findings align with Brunes et al. (2022), who observed that weight gain strongly predicts sexual precocity in Nellore heifers, supporting that animals with superior growth may reach earlier sexual maturity under favourable conditions, and Johnston et al. (2009), who reported that feed efficiency traits and nutritional environment affect heifers' age at puberty in Brahman cattle.

While the biological relevance of RG is clear, it is important to note that, as residual traits derived from conditional distributions, RG and RFI do not provide additional genetic information beyond their component traits and do not alter the theoretical selection goal when an index with appropriate economic weights is applied (Kennedy et al. 1993). Nevertheless, RG remains biologically meaningful, providing a direct measure of metabolic efficiency independent of body size (Santana et al. 2014; Ceacero et al. 2016) and allowing identification of animals that deviate from expected growth paths (Arthur et al. 2001; Bonamy et al. 2019). In practice, residual traits such as RG and RFI allow for more targeted selection on growth and feed efficiency, reducing but not eliminating correlations with other traits, which can be advantageous when constructing precise multi‐trait economic indices is challenging (Berry and Crowley 2013).

Similar trends were reported by Brunes et al. (2021), with low genetic correlations between RG and growth, feed efficiency and carcass traits in Nellore cattle. Our study adds that RG shows a moderate positive genetic correlation with W450, indicating that animals with higher RG tend to reach greater AW. The moderate positive phenotypic correlation with ECP further suggests that favourable management promoting weight gain may also advance sexual precocity.

## 4 Conclusion

Despite its low heritability, RG is a largely independent trait and shows sufficient genetic variability to respond to selection in Nellore cattle. Using it as a selection criterion can enhance feed efficiency without negatively impacting other economically important traits. However, due to the positive genetic correlation with W450 and AW, selection for RG is best positioned for intensive finishing systems where nutritional deficits are absent. In such scenarios, the economic benefits of improved gain and efficiency outweigh the potential disadvantages of increased maintenance energy requirements.

## Funding

The authors thank the Fundação de Amparo à Pesquisa do Estado de São Paulo (FAPESP, Brazil) for financial support through a PhD scholarship in Brazil granted to the first author (grant number #2023/13805–9). This study was also partially financed by the Conselho Nacional de Desenvolvimento Científico e Tecnológico (CNPq, grant number #141603/2023–2), and the Coordenação de Aperfeiçoamento de Pessoal de Nível Superior (CAPES, Brazil)—Finance Code 001.

## Conflicts of Interest

The authors declare no conflicts of interest.

## Data Availability

The data analysed in this study were obtained from the National Association of Breeders and Researchers (ANCP). The phenotypic and genotypic information was provided to the authors for academic research purposes only. The following restrictions apply: the dataset is not publicly available, and its use requires formal authorization. Requests to access these datasets should be directed to Dr. João Carlos G. Giffoni Filho, President of ANCP (email: presidencia@ancp.org.br).
